# Diagnostic Value of Hepatic Mast Cell Concentration (MCC) in NAFLD and NASH Severity and Fibrosis Grade

**DOI:** 10.30699/ijp.2024.2016320.3216

**Published:** 2024-04-29

**Authors:** Mahshid Panahi, Nasser Rakhshani, Alireza Sarhaddi, Monavvar Afzalaghaee, Hamid Rezvani, Nikoo Emtiazi, Farkhonde Sarhaddi

**Affiliations:** 1 Department of Pathology, School of Medicine, Iran University of Medical Sciences, Tehran, Iran; 2 Gastrointestinal and Liver Diseases Research Center, Iran University of Medical Sciences, Tehran, Iran; 3 Department of Educational Sciences, Farhangian University, Zahedan, Iran; 4 Social Determinant of Health Research Center, Mashhad University of Medical Sciences, Mashhad, Iran; 5 Department of Adult Hematology Oncology, School of Medicine, Shahid Beheshti University of Medical Sciences, Tehran, Iran; 6 Department of Pathology, School of Medicine, Zahedan University of Medical Sciences, Zahedan, Iran; 7 Faculty of Medical Sciences, Zahedan Branch, Islamic Azad University, Zahedan, Iran

**Keywords:** Hepatic fibrosis, Immunohistochemistry, Non-alcoholic fatty liver disease (NAFLD), Non-alcoholic steatohepatitis (NASH)

## Abstract

**Background & Objective:**

Mast cells play a role in the immune responses to fatty liver disease. The present study aimed to investigate the diagnostic value of hepatic mast cell concentration (MCC) in NAFLD and NASH severity and fibrosis grade.

**Methods:**

The present cross-sectional unremarkable hepatic histology, NAFLD, or NASH cases were enrolled. Demographic variables, BMI, hepatic stiffness assessed using fibroscan, portal inflammation, hepatic disease grade assessed using the NAFLD Activity Score (NAS), and hepatic fibrosis severity assessed using the NASH fibrosis stage, hepatic necrosis severity, and hepatic steatosis severity of the patients were collected. The hepatic specimens underwent immunohistochemical (IHC) staining.

**Results:**

Of a total of 92 patients with a mean age of 38.7±13.3 years, 56 (60.9%) were males. There were significant relationships between the NAS score of the patients and hepatic steatosis. Moreover, the NASH fibrosis stage had significant relationships with the variables of hepatic necrosis, steatosis, and stiffness. There were significant positive correlations between the mast cell concentration (MCC) in all zones of the hepatic tissue (zone 1, zone 2, zone 3, portal area, and total) and the variables of age, BMI, and hepatic necrosis, steatosis, and stiffness. The patients with a higher NASH fibrosis stage showed a significantly higher MCC in all zones of the hepatic tissue.

**Conclusion:**

Hepatic mast cell number may have a significant impact on the grade and fibrosis in NAFLD. However, it is recommended to perform further studies with larger sample sizes on patients with various etiologies for hepatic injury to confirm the present study results.

## Introduction

Obesity and metabolic syndrome have become increasingly prevalent around the world, leading to an increased incidence of diseases induced by obesity, including Non-Alcoholic Steatohepatitis (NASH) and Non-Alcoholic Fatty Liver Disease (NAFLD) (1, 2). According to the World Gastroenterology Organization in 2012, the global prevalence of NAFLD has doubled over the last two decades. Moreover, about 34% of the individuals in the United States have a type of NAFLD (3, 4). However, this prevalence is now estimated to be higher, reaching a rate of 40% (4, 5). Histologically, NAFLD, which is a benign condition, is characterized by macrovesicular steatosis, defined as the presence of hepatocytes with a single and large fat vacuole displacing the cellular nucleus (4, 6). In contrast, NASH, which has a more severe prognosis, is characterized by microvesicular steatosis and damaged hepatocytes with several small lipid vesicles not displacing the nucleus (7, 8). Moreover, NASH is usually associated with hepatic inflammation, biliary senescence, and ductular reaction, which positively correlate with hepatic fibrosis (9, 10).

The underlying mechanisms of the transition from NAFLD to NASH are not clearly illustrated (11, 12). It is estimated that insulin resistance may lead to fat deposition in the hepatocytes, which subsequently leads to hepatocyte damage, inflammation, and hepatic fibrosis (11, 13). Moreover, some factors, such as lipotoxicity, abnormal lipid metabolism, oxidative stress, genetic predisposition, altered cytokine and adipokine production, mitochondrial dysfunction, endoplasmic reticulum stress, and intestinal dysbiosis may increase the risk of NAFLD progression to NASH (11, 14).

Mast cells, which are derived from basophils, have been reported to play a role in the innate immune responses leading to hepatic injury (15, 16). Mast cells are highly involved in response to hepatic fibrosis induced by chronic inflammation, which is potentially due to sinusoidal capillarization (17, 18). This phenomenon is defined as the transformation of hepatic sinusoids into continuous capillaries (15, 17). According to animal studies, the number of mast cells has a positive correlation with the severity of hepatic fibrosis in rats with hepatic cirrhosis induced by carbon tetrachloride (18, 19). These cells produce fibrogenic cytokines, such as the basic Fibroblast Growth Factor (bFGF), Transforming Growth Factor β (TGF-β), and Platelet-Derived Growth Factor (PDGF) (19, 20). Moreover, it has been shown that mastocytosis in humans can be associated with portal hypertension and hepatic fibrosis (21, 22).

According to the studies, mice with genetic mutations leading to impaired mast cell maturation cannot become obese with an obesity-inducing diet. Moreover, they tend to have decreased levels of macrophages, chemokines, and inflammatory cytokines in the adipose tissue, which further confirms the role of mast cells in mediating the inflammatory processes (21, 23). Also, mast cells can secrete several pro-inflammatory molecules, including lipid mediators, histamine, proteoglycans, proteases (carboxypeptidases, tryptases, and chymases), and cytokines (IL-10, IL-4, IL-13, IL-5, IL-1, and TNF-α), leading to the attraction of inflammatory cells and subsequent inflammation, scarring, and fibrosis (24, 25).

Despite the evidence from animal studies on the relationship between mast cells and hepatic fibrosis, limited studies have investigated such a relationship in human models (26, 27). Therefore, the present study aimed to investigate the potential relationships between the number of mast cells in different zones of hepatic tissue and the hepatic disease grade, hepatic fibrosis stage, and other demographic, clinical, and histopathological characteristics in a group of patients with normal hepatic histology, NAFLD, or NASH in their hepatic biopsies. 

## Material and Methods

**Participant Enrollment**

The present cross-sectional study included 92 patients who presented to the Gastroenterology Clinic and Pathology Department of the Firoozgar and Mehr Hospitals, affiliated to Iran University of Medical Sciences, Tehran, Iran, underwent hepatic biopsy for suspected hepatic problems and were diagnosed with unremarkable hepatic histology, NAFLD, or NASH from October 2017 to October 2019. Demographic, clinical, and histopathological data of the patients were collected in a researcher-made questionnaire using their medical records. Moreover, the patients with autoimmune hepatitis, those with a history of viral hepatitis (hepatitis B or C), and those with a history of alcohol consumption were excluded from the present study. The normal group was collected based on other disease indications for biopsy and their liver biopsy results to compare with other groups in this study. 

**Study Variables**

Study variables included Body Mass Index (BMI), gender, age, hepatic stiffness assessed using fibroscan, hepatic fibrosis severity assessed using the NASH fibrosis stage, hepatic disease grade assessed using the NAFLD Activity Score (NAS), portal inflammation, hepatic necrosis severity, and hepatic steatosis severity as shown in Table 1 (28). The BMI, gender, age, and fibroscan results were obtained from the medical records of the patients. Moreover, the NASH fibrosis stage, the NAS score, portal inflammation, hepatic necrosis severity, and hepatic steatosis severity were obtained from the pathology reports of the patients.

**Table 1:** NAFLD Activity Score (NAS) and NASH fibrosis stage.

Histological feature	Score	Description
Steatosis	0	<5%
	1	5%-33%
	2	33%-66%
	3	>66%
Hepatocyte Ballooning	0	None
	1	Few
	2	Many
Lobular Inflammation	0	0-1 foci per ×400 field
	1	2-3 foci per ×400 field
	2	4-5 foci per ×400 field
	3	>5 foci per ×400 field

**Abbreviations:** NASH: Non-Alcoholic Steatohepatitis

**Mast Cell Detection and Recording**

The paraffin blocks made from the hepatic specimens of the patients were cut using a microtome and underwent immunohistochemical staining for tryptase to detect the mast cells in the hepatic tissue. The slides were evaluated by three independent pathologists using a light microscope. Each pathologist counted the mast cell numbers in the peripheral zone (Zone 1), intermediate zone (Zone 2), central zone (Zone 3), and portal area of the hepatic parenchyma in five fields at ×400 magnification and recorded the findings. 

**Statistical Analysis**

The significance of the relationships between the study variables was investigated using the one-way Analysis of Variance (ANOVA), independent sample t-test, Kruskal Wallis test, Spearman’s correlation coefficient, and Fisher’s exact test. Moreover, the categorical variables, normally distributed continuous variables, and non-normally distributed continuous variables were described using the frequency and percentage (%), mean ± Standard Deviation (SD), and median and Interquartile Range (IQR), respectively. The confidence interval was considered at 95%. Finally, the statistical analysis was performed using the IBM SPSS software version 25 (SPSS Inc., Chicago, Ill., USA), and the significance level was set at 0.05.

## Results

According to our findings, the study participants included 56 (60.9%) males, with a mean age of 38.7±13.3 years. Out of a total of 92 patients whose hepatic specimens were used for the present study, 10 had normal hepatic histology, 5 had NAFLD, and 77 were suffering from NASH. Moreover, there was no significant relationship between age and final diagnosis of the patients (*P*>0.05). However, the patients with NAFLD had the highest BMI, while those with normal hepatic histology had the lowest BMI, showing significant differences (*P*<0.05) (Table 2).

**Table 2:** Relationships between mast cell concentration in different zones of the hepatic tissue and study variables.

		Normal (n=10)	NAFLD (n=5)	NASH (n=77)	P-value
Age	Mean ± SD	35.50±13.27	43.20±14.91	38.83±13.27	0.565*
Gender; N (%)	Male	5(50.0)	4(80)	47(61)	0.56**
	Female	5(50)	1(20)	30(39)	
BMI	mean ± SD	23.11±3.45	30.45±1.95	28.9±4.61	0.001*
Hepatic steatosis	mean ± SD	0.2±0.63	25.60±9.96	50.19±20.34	0.001***
Liver stiffness (fibroscan) N (%)	F0-F1	6(85.7)	1(25)	19(35.8)	-
	F2	1(14.3)	0(0.0)	9(17.0)	
	F3	0(0.0)	2(50.0)	13(24.5)	
	F4	0(0.0)	1(25.0)	12(22.6)	
Necrosis; N (%)	None	10(100)	3(60)	28(36.4)	-
	Few Mild	0(0.0)	2(40)	44(57.1)	
	Moderate	0(0.0)	0(0.0)	5(6.5)	
Grade	Normal	10(100)	0(0)	0(0)	-
	2	0(0)	2(40)	3(3.9)	
	3	0(0)	2(40)	12(15.6)	
	4	0(0)	1(2)	24(31.2)	
	5	0(0)	0(0)	24(31.2)	
	6	0(0)	0(0)	11(14.3)	
	7	0(0)	0(0)	3(3.9)	
Stage	0	10(100)	2(40)	19(24.7)	**-**
	1	0(0)	2(40)	16(20.8)	
	2	0(0)	0(0)	21(27.3)	
	3	0(0)	1(20)	13(16.9)	
	4	0(0)	0(0)	8(10.4)	

*one-way ANOVA

**exact t test

*** Kruskal Wallis

There was a significant relationship between the NAS score of the patients and hepatic steatosis (*P*<0.05). Therefore, the patients with a higher NAS score showed significantly higher hepatic steatosis and stiffness. However, no significant relationship was found between NAS score and any of the variable’s age or portal inflammation (both *P*>0.05).

Moreover, the NASH fibrosis stage had significant relationships with the variables of hepatic necrosis, steatosis, and stiffness (*P*<0.05). Therefore, the patients with a higher NASH fibrosis stage had significantly higher hepatic steatosis, necrosis, and stiffness. However, there was no significant relationship between the NASH fibrosis stage and any of the variable’s age or portal inflammation (*P*>0.05).

**Fig. 1:**
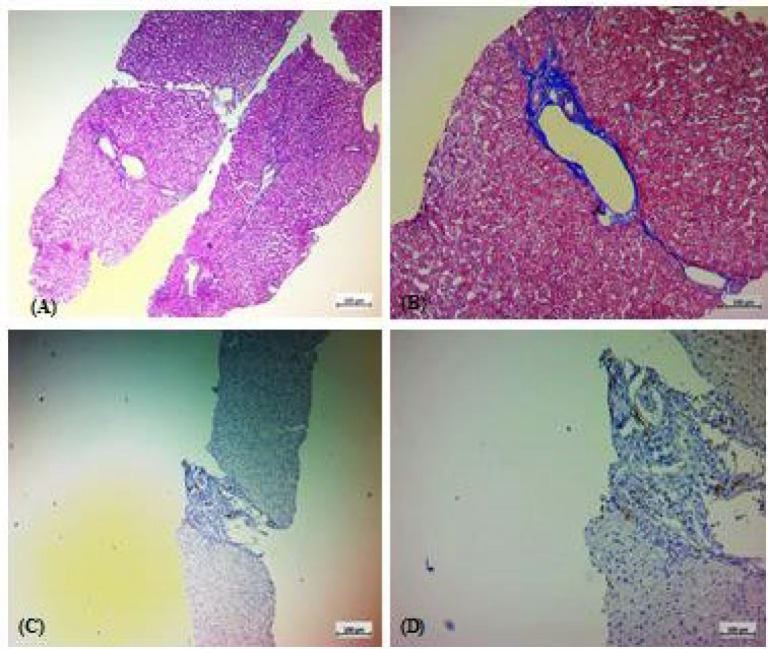
Histologic features of a hepatic specimen from a case with normal hepatic histology. A: Hematoxylin and Eosin (H & E) staining. B: Trichrome staining. C & D: immunohistochemical staining for tryptase.

**Fig. 2:**
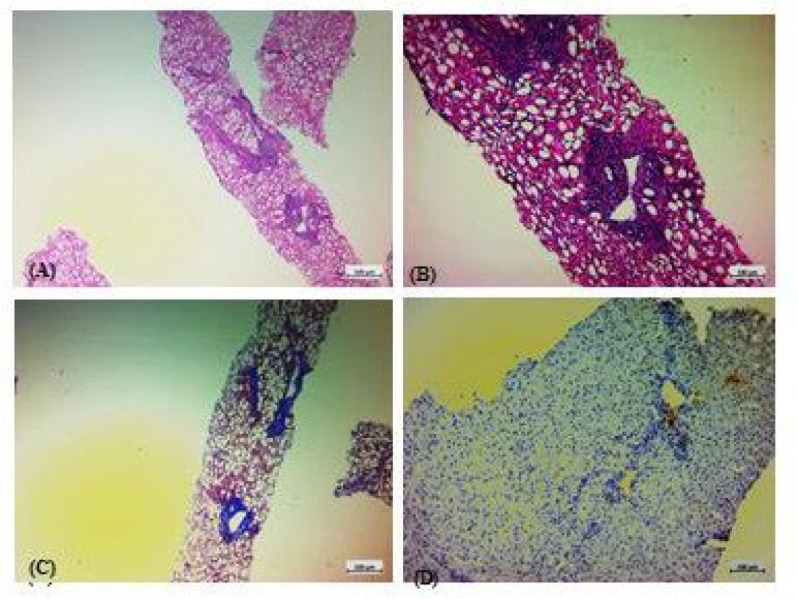
Histopathologic features of a case with non-alcoholic steatohepatitis (NASH), with a NAS score of 5/8 and a NASH fibrosis stage of 0/4. A&B: Hematoxylin and Eosin (H & E) staining. C: Trichrome staining. D: immunohistochemical staining for tryptase.

**Fig. 3:**
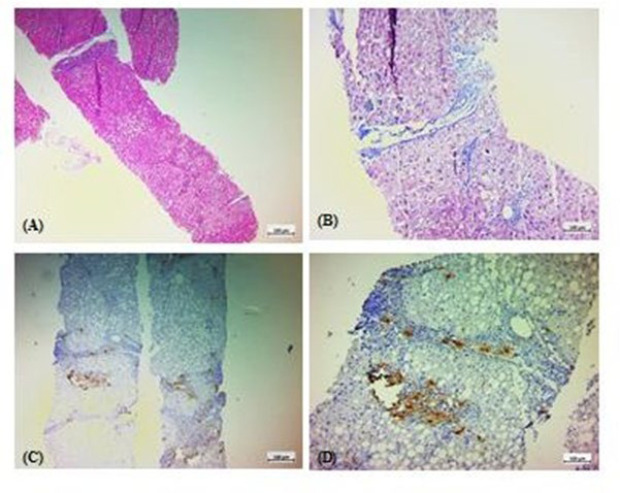
Histopathologic features of a case with impeding cirrhosis, mostly due to NASH, with a NAS score of 5/8 and a NASH fibrosis stage of 3/4. A: Hematoxylin and Eosin (H & E) staining. B: Trichrome staining. C & D: immunohistochemical staining for tryptase.

**Fig. 4:**
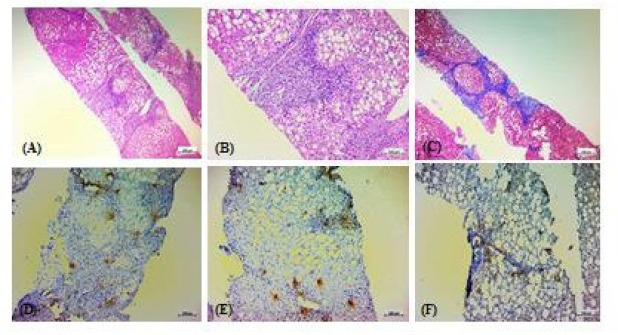
Histopathologic features of a case with impending cirrhosis due to NASH, with a NAS score of 5/8 and a NASH fibrosis stage of 4/4. A & B: Hematoxylin and Eosin (H & E) staining. C: Trichrome staining. D, E, &F: immunohistochemical staining for tryptase.

According to our findings, there were significant positive correlations between the mast cell concentration (MCC) in all zones of the hepatic tissue (Zone 1, Zone 2, Zone 3, portal area, and total) and the variables of age, BMI, and hepatic necrosis, steatosis, and stiffness (all *P*<0.05) (Table 3). The relationships between the MCC in different zones of the hepatic tissue and the study variables are presented in Figures 1, 2, 3, and 4.

As our findings determine, there were significant and positive relationships between the MCC in all zones and the hepatic disease grade assessed using the NAS score (all *P*<0.05). The relationships between the MCC in different zones of the hepatic tissue and the hepatic disease grade are presented in Table 4 in detail and in Figure 1. Also, MCC in all zones and the hepatic fibrosis were strongly and significantly correlated (all *P*<0.001). Therefore, the patients with higher NASH fibrosis stage demonstrated a significantly higher MCC in all zones of the hepatic tissue. Relationships between the MCC in different zones of the hepatic tissue and hepatic fibrosis are presented in Table 5 in detail.

**Table 3:** Mast cell concentration (MCC) median in different zones of the hepatic tissue

		Normal (n=10)	NAFLD (n=5)	NASH (n=77)	P-value*
Mast cell concentration median (IQR)	Zone 1	0.1 (0 to 0.3)	1 (0.4 to 1.7)	0.8 (0.4 to 1.8)	0.0001
	Zone 2	0.2 (0.2 to 0.25)	0.4 (0.3 to 1.3)	0.6 (0.2 to 1.2)	0.03
	Zone 3	0 (0 to 0)	0 (0 to 0.6)	0.2 (0 to 0.8)	0.005
	Portal area	0.2 (0.0 to 0.45)	0.6 (0.4 to 2.1)	1 (0.6 to 2.2)	0.0001
	Total	0.6 (0.2 to 1.1)	2 (1.3 to 5.5)	2.4 (1.4 to 5.9)	0.0001

* Kruskal Wallis was used

**Table 4:** Relationship between hepatic disease grade (assessed using the NAS score) and mast cell concentration (MCC) in different zones of the hepatic tissue.

NAS score	MCC groups	Zone 1	Zone 2	Zone 3	Portal area	Total	p-value
2/8	NASH	0.8 (0.2 to 0.8)	0.6 (0 to 0.6)	0.2 (0 to 0.2)	0.8 (0.4to0.8)	2.4 (0.8 to 2.4)	0.17
	NAFLD	1.2 (1 to 1.2)	0.4 (0.4 to 0.4)	0.2 (0 to 0.2)	0.7 (0.2to0.7)	2.5 (2 to 2.5)	0.02
3/8	NASH	0.4 (0.2 to 0.4)	0.2 (0.05 to 0.4)	0 (0 to 0.15)	0.6 (0.6to0.8)	1.4 (0.8 to1.55)	<0.0001
	NAFLD	1.2 (0.4 to 1.2)	1.2 (0.2 to 1.2)	0.4 (0 to 0.4)	1.8 (0.6to1.8)	4.6 (1.2 to 4.6)	0.11
4/8	NASH	0.6 (0.4 to 1.45)	0.5 (0.4 to 1.05)	0.2 (0 to 0.7)	0.8 (0.45to2.15)	2.2 (1.25 to 5.25)	<0.0001
	NAFLD	-	0.4 (0.4 to 0.4)	-	0 (0 to 0)	-	
5/8	NASH	0.8 (0.6 to 1.85)	0.6 (0.4 to 1.2)	0.3 (0.2to0.6)	1.2 (0.85to2.25)	3 (1.95 to 5.2)	<0.0001
	NAFLD	-	-	-	-	-	
6/8	NASH	1.8 (0.8 to 3)	2 (0.8 to 2.2)	1 (0.4to1.6)	2.4 (1.2 to 3)	7.2 (3.4to10.4)	<0.0001
	NAFLD	-	-	-	-	-	
7/8	NASH	1.8 (0.6 to 1.8)	1 (0.6 to 1)	0.8 (0.2 to0.8)	2 (0.8 to 2.1)	5.6 (2.2to5.6)	0.02
	NAFLD	-	-	-	-	-	

MCC: mast cell concentration; NAS: NAFLD Activity Score

**Fig 5:**
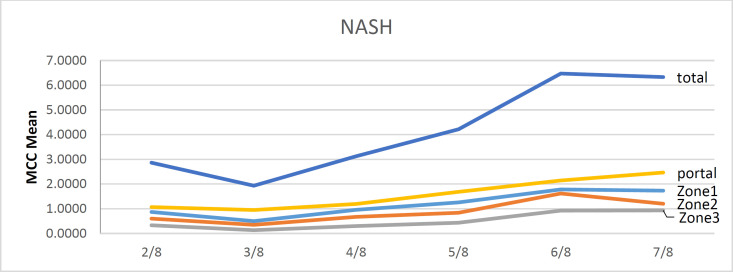
Diagram of MCC in the NASH cases (n=77). MCC: mast cell concentration.

**Table 5:** Relationship between hepatic fibrosis (assessed using the NASH fibrosis stage) and mast cell concentration (MCC) in different zones of the hepatic tissue.

NASH fibrosis stage MCC	Normal	0/4	1/4	2/4	3/4	4/4	P-value*
Zone 1	0.1 (0.3)	0.4 (0.2)	0.6 (0.4)	0.8 (0.5)	2.2 (1.1)	2.6 (1.3)	< 0.001
Zone 2	0.2 (0.05)	0.2 (0.4)	0.4 (0.25)	0.6 (0.5)	1.9 (0.9)	2.0 (0.35)	< 0.001
Zone 3	0 (0)	0 (0)	0 (0.2)	0.2 (0.2)	0.8 (0.5)	1.3 (0.65)	< 0.001
Portal area	0.2 (0.45)	0.4 (0.2)	0.8 (0.4)	1.2 (0.8)	3.0 (1.25)	3.2 (2.15)	< 0.001
Total	0.6 (0.9)	1.0 (0.8)	2.0 (1.0)	3.0 (1.2)	7.8 (4.1)	9.0 (3.15)	< 0.001

* Kruskal Wallis was used

## Discussion

The present study investigated the relationship between each of the variables’ hepatic disease grade, hepatic fibrosis, and mast cell concentration with various demographic, clinical, and histopathological characteristics of the patients. Moreover, we thoroughly investigated the relationship between mast cell concentration with hepatic disease grade and hepatic fibrosis. In this regard, we obtained a concentration of mast cells in different zones of the hepatic tissue and compared them with the hepatic disease grade (assessed using the NAS scoring), hepatic fibrosis (assessed using the NASH fibrosis staging), and several other patient-related factors in three groups of NAFLD, NASH and normal hepatic biopsies. According to our findings, out of a total of 92 patients, there were significant positive correlations between the NAS score and the variables of hepatic steatosis and stiffness. Also, significant positive correlations were observed between the NASH fibrosis stage and hepatic necrosis, steatosis, and stiffness. Additionally, there were significant positive correlations between the total concentration of mast cells and the variables of age, BMI, portal inflammation, hepatic necrosis, steatosis, and stiffness. Moreover, the patients diagnosed with NASH showed the highest concentration of mast cells, while those with normal hepatic histology demonstrated the lowest number of mast cells, indicating a significant difference. Finally, there were significant positive correlations between the concentration of the mast cells in all regions and the variables of NASH fibrosis stage and NAS score. 

To the best of our knowledge, limited studies have ever investigated the concentration of mast cells in the hepatic tissue of patients with NAFLD and NASH (29). A study by Lombardo* et al.* (27) investigated the concentration of the mast cells in the parenchymal and periportal regions of 106 hepatic tissue specimens obtained from patients with unremarkable hepatic histology, NAFLD, and NASH. The authors reported a significant difference between specimens with unremarkable hepatic histology and those with NASH fibrosis stages 3-4 in the concentration of periportal, parenchymal, and total mast cells, while mast cell concentrations were not different between those with unremarkable hepatic histology and the patients with NAFLD. Moreover, the patients with unremarkable hepatic histology (supplementary figures 1-4) and those with NASH fibrosis stage 0-1 and 2 showed significant differences in parenchymal mast cell concentration. Also, the patients with NASH fibrosis stage 3-4 had significantly higher mast cell concentration in parenchymal and periportal areas and in total compared to the patients with NASH fibrosis stages 0-1 and 2.

The findings of the mentioned study were compatible with ours; both showed a significantly higher concentration of mast cells in all regions of the hepatic tissue in the patients with NASH compared to those with NAFLD or unremarkable hepatic histology. However, the present study also investigated the relationship between mast cell concentration and several patient-related factors, such as NAS score, NASH fibrosis stage, final diagnosis, age, BMI, portal inflammation, hepatic necrosis, steatosis, and stiffness. The study by Lombardo* et al.* only investigated the relationship between mast cell concentration and hepatic fibrosis and the final diagnosis of the patients (27).

Another study, which was conducted by Armbrust* et al.*, (30) evaluated mast cell distribution in the hepatic specimens from human and rat models with unremarkable hepatic histology, hepatic injury without fibrosis, and fibrotic hepatic injury, showing the rare number of mast cells in portal areas of the normal hepatic tissue in both humans and rats. Moreover, the number of mast cells was insignificantly increased in the hepatic tissue with non-fibrotic damage compared to the normal tissue. However, fibrotic specimens of both species contained significantly elevated numbers of mast cells in the fibrous septa and portal tracts.

The above findings were compatible with ours in showing the significantly elevated number of mast cells in the human hepatic tissue with fibrotic damage, as well as an insignificant difference between the normal hepatic tissue and tissue with non-fibrotic injury. However, the present study only investigated the human models with unremarkable hepatic histology, NASH, and NAFLD. Moreover, we studied potential relationships between the number of mast cells and several patient-related factors. However, the study by Armbrust* et al.* (30) investigated both humans and rats. Moreover, the hepatic specimens from human models were obtained from patients with various problems, such as alcoholic liver diseases and viral hepatitis, while the present study excluded patients with alcohol consumption and viral or autoimmune hepatitis.

A study by Lewandowska* et al.* (21) investigated number of the mast cells in different areas of the hepatic tissue in specimens from obese patients with NAFLD and compared the numbers with the control group, showing a significantly higher number of mast cells in obese patients with NAFLD, especially in the fibrous septa and portal areas. Moreover, the authors found a significant positive correlation between hepatic fibrosis and number of the mast cells in the fibrous septa and portal areas. However, this finding was not true for several mast cells in the hepatic lobules. Also, there was a significant relationship between portal inflammation and mast cell numbers.

The findings of the above study were compatible with our findings in a significant positive correlation between hepatic fibrosis and number of the mast cells. However, the mentioned study did not find a significant relationship between the mast cell numbers in the hepatic lobules and hepatic fibrosis, while our study reported a significant difference. Moreover, the study by Lewandowska* et al.* (21) reported a significant relationship between hepatic inflammation and mast cell numbers, which was not reported in our study.

Our study had some limitations as well. The present study excluded all the patients with viral or autoimmune hepatitis and alcohol consumption, narrowing the study population to those with normal hepatic histology, NASH, and NAFLD. However, similar studies included patients with hepatic injury due to several different pathologies, such as alcoholic liver disease, viral hepatitis, and autoimmune hepatitis. Moreover, the present study only investigated a limited number of patients with normal hepatic histology, NAFLD, or NASH.

## Conclusion

In general, the present study concluded that there may be significant relationships between number of the mast cells in all zones of the hepatic tissue and t grade and fibrosis in NASH. Moreover, the number of mast cells may be significantly affected by age, BMI, portal inflammation, hepatic necrosis, steatosis, and stiffness. Also, portal inflammation may be associated with no significant effect on mast cell number, NAS score, or NASH fibrosis stage. Finally, it is recommended to perform further studies with larger sample sizes and more patient-related variables on patients with hepatic injury due to various etiologies, to confirm the results of the present study.
